# Validez de la radiografía periapical digital y la tomografía computarizada de haz cónico en la detección de defectos óseos peri-implantarios: estudio *in vitro*

**DOI:** 10.21142/2523-2754-1101-2023-141

**Published:** 2023-03-26

**Authors:** César C Elera Morales, Carmen T Castro Ruiz

**Affiliations:** 1 Carrera de Estomatología, Universidad Científica del Sur. Lima, Perú. eleracesar4@gmail.com, ccastro@cientifica.edu.pe Universidad Científica del Sur Carrera de Estomatología Universidad Científica del Sur Lima Peru eleracesar4@gmail.com ccastro@cientifica.edu.pe

**Keywords:** periimplantitis, tomografia computarizada de haz cónico, implantes dentales, radiografía dental, radiolucencia periimplantar, peri-implantitis, cone beam computed tomography, dental implants, dental radiography, peri-implant radiolucency

## Abstract

**Objetivo:**

Comparar la precisión diagnóstica de la radiografía digital directa (RDD) y la tomografía computarizada de haz cónico (TCHC) en la detección de defectos óseos periimplantarios.

**Materiales y métodos:**

Los implantes se colocaron en 5 costillas bovinas frescas (3 sin defectos óseos periimplantarios, 12 con defectos óseos periimplantarios de 1.4mm) y se tomaron imágenes usando (i) sistema de rayos x portátil (Dexcowin 3000), (ii) sensor intraoral de rayos x (Sensor H1/Sensor H2), (iii) TCHC de volumen limitado con 3D Accuitomo 80 (Castellini). Las imágenes de cada uno se presentaron aleatoriamente a 10 examinadores. La confianza en el diagnóstico de la presencia o ausencia de una radiotransparencia periimplantaria se registró en una escala de defecto óseo: definitivamente ausente, dudas sobre el defecto si está ausente o presente, defecto definitivamente presente. Se realizó el análisis inter e intraexaminador mediante una prueba de kappa.

**Resultados:**

Se ha obtenido una coincidencia de un 83,3% entre la radiografía digital directa y la tomografía computarizada de haz cónico en la pieza A, de un 100% en la pieza B y de un 88% en la pieza C, lo que da un total promedio de coincidencia del 90.43%.

**Conclusión:**

La radiografía digital directa brindó un mismo resultado que una tomografía computarizada de haz cónico en la detección de defectos óseos periimplantares en una fase inicial. Las radiografías digitales son un método fiable y válido y funcionan significativamente mejor que la tomografía computarizada de haz cónico para detectar defectos óseos periimplantarios en una fase inicial.

## Abstract (en)

**Abstract:**

To compare the diagnostic accuracy of digital radiography (DR) and cone beam computed tomography (CBCT) in the detection of peri-implant bone defects.

**Materials and methods:**

Implants were placed in 5 fresh bovine ribs (3 without peri-implant bone defects, 12 with 1.4mm peri-implant bone defects) and images were taken using (i) portable x-ray system (DEXCOWIN 3000), (ii) intraoral x-ray sensor (SENSOR H1/SENSOR H2), (iii) volume-limited HSCT with 3D Accuitomo 80 (CASTELLINI). Images of each were randomly presented to 10 examiners. Confidence in the diagnosis of the presence or absence of a peri-implant radiolucency was recorded on a scale of bone defect definitely absent, doubts about the defect if absent or present, defect definitely present. Analyzes was performed using a kappa test.

**Results:**

There has been a coincidence between the direct digital radiography and the cone beam computed tomography in piece A of 83.3%, in piece B of 100% and in piece C of 88%, giving an average total coincidence 90.43%.

**Conclusion:** Direct digital radiography provided the same results as cone beam computed tomography in the detection of peri-implant bone defects at an early stage. Digital radiographs are a reliable and valid method and perform significantly better than cone beam computed tomography for detecting peri-implant bone defects at an early stage.

## INTRODUCCIÓN

El uso de implantes dentales se ha convertido en un método frecuente y eficaz para sustituir los dientes ausentes ^1^^-^^3^. La alta predictibilidad de las técnicas quirúrgicas empleadas y su baja incidencia de fracasos han hecho que se considere como una de las primeras opciones de tratamiento en la rehabilitación oral ^4^^-^^6^.

El 2% de los implantes fracasan por diversas razones, entre ellas las enfermedades periimplantarias ^7^^,^^8^. Para facilitar su detección y diagnóstico, han sido publicadas diversas clasificaciones de defectos óseos periimplantarios. Monje *et al*. (2019) utilizaron la tomografía computarizada de haz cónico (TCHC) para clasificar la topografía de los defectos óseos periimplantarios en tres clases principales, según el número de paredes restantes y la extensión de la pérdida ósea vertical. Informaron que la morfología del defecto más frecuente entre los casos de periimplantitis era un defecto infraóseo de dos o tres paredes tipo Ib (87% de los casos), que se presentaba con dehiscencia bucal ^9^^-^^11^.

Diversos estudios, como los de Eberhard *et al*. ^12^, Renvert *et al*. ^13^ y Gulati *et al*. ^14^, concuerdan en que el control deficiente de la placa dental ha sido identificado como un factor de riesgo potencial para el desarrollo de la periimplantitis. Por consiguiente, proponen un mantenimiento de rutina conocido como terapia de higiene de implante (THI), el cual se realiza luego de haber sido rehabilitado el implante. La THI consiste en visitar al dentista cada 3 meses durante el primer año y que cada cita de mantenimiento dure 1 hora aproximadamente para realizar un sondaje periodontal con una fuerza no mayor a 0,25 N. En los casos en los que se requiere, se toman radiografías periapicales utilizando la técnica de paralelismo de cono largo para evaluar el nivel óseo y descartar defectos. En este caso de rutina, se utilizan las radiografías periapicales digitales (RD). Si se observan signos clínicos como sangrado o supuración, profundidad al sondaje aumentada y el diagnóstico radiográfico no es suficiente, se indica una tomografía computarizada de haz cónico (TCHC) ^12^^-^^14^.

Las RD están siendo cada vez más utilizadas y sus principales ventajas son la menor dosis de radiación para el paciente, las herramientas de medición que nos brindan, la obtención rápida de la imagen, su reutilización, el menor empleo de aparatología y su fácil uso. Mediante programas informáticos, se pueden modificar el contraste, el brillo, la escala de grises, a fin de obtener una mejor resolución ^15^^-^^19^.

Dentro de la radiología digital encontramos dos métodos ^15^. La radiografía digital directa (RDD) no requiere ningún tipo de escaneado luego de ser expuesta a los rayos X, sino que el propio sistema informático se encarga de obtener la imagen. Este método emplea como receptor de los rayos X a un captador rígido conectado a un cable, a través del cual se envía la información a un ordenador. La radiografía digital indirecta (RDI) captura la imágen de forma analógica en una placa de fósforo fotoestimulable y es convertida en digital después de haber sido procesada y escaneada.

Tanto la radiografía digital directa como la indirecta poseen la limitación de ser bidimensionales, y presentar distorsión, proyecciones y superposición de imagen. El avance de nuevos sistemas de imágenes ha llevado al desarrollo de tecnologías como los datos de adquisición de volumen, que han permitido el incremento de la proyección detallada en un medio 3D. La tomografía de haz cónico (THC) puede adquirir y procesar datos para presentar modelos reconstruidos a través de imágenes transversales de planos axiales, coronales o sagitales. Para ello, utiliza un cono en forma de haz de rayos X activado en una sola rotación, que le permite almacenar los datos de la proyección volumétrica de los diferentes tipos de detectores bidimensionales a través de un *software*. 

Por otro lado, respecto de los métodos radiográficos para la detección y el seguimiento de los defectos óseos periimplantarios, se debe tener en cuenta que para su uso debe haber una fuerte indicación clínica, además del principio de ALARA, que hace referencia a una baja dosis de radiación para el paciente (tan baja como sea posible) ^20^.

Con respecto a la periimplantitis, se ha reportado una baja tasa de éxito en el tratamiento, en especial en las etapas más avanzadas de la enfermedad, lo que termina en la pérdida del implante dental. Por esa razón, se ha descrito la importancia de la etapa de mantenimiento para una detección temprana de esta enfermedad y una solución oportuna para las complicaciones alrededor de los implantes dentales ^13^.

La evaluación radiológica de los implantes dentales es un procedimiento habitual en la práctica clínica diaria; sin embargo, la validación del uso clínico de estos instrumentos radiográficos en el diagnóstico y tratamiento de defectos óseos periimplantares es limitado en nuestro medio. En consecuencia, es importante saber que tan válidas son este tipo de técnicas imagenológicas para la detección de defectos óseos periimplantarios, en especial de los más frecuentes encontrados en la periimplantitis. El objetivo de esta investigación fue comparar la precisión diagnóstica de la radiografía digital directa (RDD) y la tomografía computarizada de haz cónico (TCHC) en la detección de defectos óseos periimplantarios.

## MATERIALES Y MÉTODOS

Este trabajo experimental fue aprobado por el comité de ética de la Universidad Científica del Sur con el número N.° 953-2021-PRE8.

Se obtuvieron y prepararon costillas de bovino frescas retirando los tejidos blandos. Se crearon cinco bloques de costilla de tamaño adecuado para contener un total de tres implantes por cada costilla. Las costillas se mantuvieron congeladas a fin de minimizar la pérdida de humedad.

La preparación del lecho receptor del implante se marcó con un lapicero azul, a una distancia de 3 mm entre implante e implante, en el borde superior de los bloques óseos. Se utilizaron implantes de conexión interna de 5 x 7,5 mm (Neo Biotech) y se crearon los defectos óseos según el diámetro y la longitud definida por Monje *et al*. Luego, se tomaron imágenes radiográficas y tomográficas, respectivamente, de los bloques óseos en la Clínica Dental Sueldo Medical Dent y en el centro radiológico IDX. 

Antes de la obtención de imágenes, los bloques óseos se hidrataron sumergiéndolos en agua. Se obtuvo imágenes de todos los implantes usando radiografía periapical digital directa (I-sensor H1 / sensor H2) y tomografía computarizada de haz cónico (Accuitomo 80 Castellini).

Los datos se analizaron utilizando el software estadístico IBM SPSS V26; asimismo, se realizó el análisis de concordancia de kappa, matriz de confusión en la que se halló los indicadores de sensibilidad y especificidad. Los VPP (valores predictivos positivos) y VPN (valores predictivos negativos) se hallaron mediante una función de puntuación de presencia y ausencia. Asimismo, las estadísticas kappa también fueron utilizadas para evaluar la concordancia entre examinadores. En este estudió se consideró que cualquier valor kappa por debajo de 0,4 indicará una concordancia deficiente, cualquier valor entre 0,41 y 0,60 se considerará una concordancia moderada, cualquier valor mayor a 0,6 se considerará una concordancia sustancial, y cualquier valor mayor a 0,8 se considerará muy buen acuerdo (Landis y Koch, 1977) ^21^.

La calibración intraobservador se realizó en dos fases: 1) sesiones de unificación de criterios entre los observadores y unificación de criterios del investigador principal; 2) la prueba piloto se realizó con tres implantes (6 superficies) a las cuales se les tomaron radiografías con los dos sistemas mencionados y, posteriormente, se prepararon para ser observados.

En la prueba piloto, se realizó la calibración interexaminador e intraexaminador de los examinadores. El piloto tuvo como objetivo disminuir la variación individual, aumentar la fiabilidad y mantener la repetitividad para garantizar la confiabilidad y validez. Con el fin de determinar la reproducibilidad de los valores de detección de los defectos óseos periimplantares, se obtuvo un valor de kappa de 0,80.

## RESULTADOS

De acuerdo con la Tabla 1, la radiografía digital directa y la tomografía computarizada de haz cónico han concordado en 50 piezas óseas, en las que existe una ausencia de defectos óseos periimplantarios definitivamente. Asimismo, han concordado en 89 piezas óseas donde el defecto óseo está presente definitivamente; por otro lado, se obtuvo un valor de kappa de 0,847, lo cual indica que existe una muy buena concordancia entre el diagnóstico de la imagen radiográfica digital directa y la tomografía computarizada de haz cónico. Además, el p-valor es menor al 5% de significancia; por lo tanto, dicha concordancia es significativa o relevante (Tabla 2). Como se puede observar en la Tabla 3, se ha obtenido una tasa de verdaderos positivos (sensibilidad) del 95%, lo que representa una gran proporción de casos positivos bien clasificados (ausencia de defecto). También se obtuvo una tasa de verdaderos negativos (especificidad) del 91%, lo cual indica una alta proporción de casos negativos bien clasificados (presencia de defecto). Con respecto a la precisión, se obtuvo un 95%, lo que representa una muy buena cercanía en la predicción del valor verdadero. Finalmente, se obtuvo una exactitud del 93%, lo que representa el porcentaje de predicciones correctas frente al total de la muestra.

**Tabla 1:** Validez diagnóstica de la imagen radiográfica digital directa y la tomografía computarizada de haz cónico en la detección de defectos óseos periimplantarios

Tomografía	Radiografía			Total
	Defecto definitivamente ausente	Dudas sobre el defecto si está ausente o presente	Defecto definitivamente presente	
Defecto definitivamente ausente	50	0	5	55
Dudas sobre el defecto si está ausente o presente	0	0	1	1
Defecto definitivamente presente	0	5	89	94
Total	50	5	95	150

**Tabla 2:** Medidas simétricas de concordancias

Indicador	Valor
Valor de kappa	0,847
Error estándar asintótico	0,043
T aproximada	11,062
Significancia	P > 0,001

**Tabla 3:** Métricas asociadas con la matriz de confusión

Métrica	Valor
Sensibilidad	0,95
Especificidad	0,91
Precisión	0,95
Exactitud	0,93

Con relación a la pieza ósea A, la radiografía digital directa y la tomografía computarizada de haz cónico han concordado en 20 piezas óseas, en las que existe una ausencia de defectos óseos periimplantarios definitivamente. Asimismo, han concordado en 25 piezas óseas, en las cuales el defecto óseo está presente definitivamente. Por otro lado, para la pieza ósea B, la radiografía digital directa y la tomografía computarizada de haz cónico han concordado en 20 piezas óseas, en las que existe una ausencia de defectos óseos periimplantarios definitivamente. Del mismo modo, han concordado en 30 piezas óseas en las cuales el defecto óseo está presente definitivamente. Finalmente, para la pieza C, han concordado en 10 piezas óseas con una ausencia de defectos óseos periimplantarios definitivamente, y han concordado en 34 piezas óseas con defectos óseos presentes definitivamente (Tabla 4). 

**Tabla 4:** Validez diagnóstica de la imagen radiográfica digital directa y la tomografía computarizada de haz cónico en la detección de defectos óseos periimplantarios

Pieza ósea	Tomografía	Radiografía			Total
		Defecto definitivamente ausente	Dudas sobre el defecto si está ausente o presente	Defecto definitivamente presente	
Pieza A	Defecto definitivamente ausente	20	0	0	20
	Dudas sobre el defecto si está ausente o presente	0	0	0	0
	Defecto definitivamente presente	0	5	25	30
	Total	20	5	25	50
Pieza B	Defecto definitivamente ausente	20	0	0	20
	Dudas sobre el defecto si está ausente o presente	0	0	0	0
	Defecto definitivamente presente	0	0	30	30
	Total	20	0	30	50
Pieza C	Defecto definitivamente ausente	10	0	5	15
	Dudas sobre el defecto si está ausente o presente	0	0	1	1
	Defecto definitivamente presente	0	0	34	34
	Total	10	0	40	50

De acuerdo con la Tabla 5, se obtuvo valores de kappa entre 0,697 y 1,000, lo que indica que existe una buena concordancia entre el diagnóstico de la imagen radiográfica digital directa y la tomografía computarizada de haz cónico. Asimismo, los p-valores obtenidos son menores al 5% de significancia; por lo tanto, dicha concordancia es significativa o relevante. 

**Tabla 5:** Medidas simétricas de concordancias

Pieza ósea	Valor de kappa	Error estándar asintótico	T aproximada	Significación aproximada
Pieza A	0,815	0,072	6,732	P > 0,001
Pieza B	1,000	0,000	7,071	P > 0,001
Pieza C	0,697	0,110	5,337	P > 0,001

Como se puede observar en la Tabla 6, se ha obtenido para la pieza ósea A una tasa de verdaderos positivos (sensibilidad) del 83%, lo que representa una gran proporción de casos positivos bien clasificados (ausencia de defecto). Además, se obtuvo una tasa de verdaderos negativos (especificidad) del 100%, lo cual significa una alta proporción de casos negativos bien clasificados (presencia de defecto). Con respecto a la precisión, se obtuvo un 100%, lo que indica una muy buena cercanía en la predicción del valor verdadero, y una exactitud del 90%, que representa el porcentaje de predicciones correctas frente al total de la muestra.

**Tabla 6:** Métricas asociadas con la matriz de confusión

Pieza ósea	Sensibilidad	Especificidad	Precisión	Exactitud
Pieza A	0,83	1,00	1,00	0,9
Pieza B	1,00	1,00	1,00	1,00
Pieza C	0,83	1,00	1,00	0,88

En el caso de la pieza ósea B, se ha obtenido una tasa de verdaderos positivos (sensibilidad) del 100%, una gran proporción de casos positivos bien clasificados (ausencia de defecto). También se obtuvo una tasa de verdaderos negativos (especificidad) del 100%, lo cual implica una alta proporción de casos negativos bien clasificados (presencia de defecto). Con respecto a la precisión, se obtuvo un 100%, lo que indica una muy buena cercanía en la predicción del valor verdadero, y una exactitud del 100%, que representa el porcentaje de predicciones correctas frente al total de la muestra. 

Por último, para la pieza ósea C, se ha obtenido una tasa de verdaderos positivos (sensibilidad) del 83%, lo que significa una gran proporción de casos positivos bien clasificados (ausencia de defecto). Asimismo, se obtuvo una tasa de verdaderos negativos (especificidad) del 100%, lo que indica una alta proporción de casos negativos bien clasificados (presencia de defecto). Con respecto a la precisión, se obtuvo un 100%, lo que implica una muy buena cercanía en la predicción del valor verdadero, y una exactitud del 88%, que representa el porcentaje de predicciones correctas frente al total de la muestra.

## DISCUSIÓN

El objetivo de esta investigación fue comparar la precisión diagnóstica de la (RDD) y la (TCHC) en la detección de defectos óseos periimplantarios, buscando recomendar al clínico la mejor herramienta para sus pruebas diagnósticas del área. Es claro que hay pocos estudios publicados respecto al tema. En este sentido está claro que las radiografías periapicales digitales convencionales son el método de imagen estándar utilizado en el seguimiento radiográfico de implantes dentales. Diversos estudios *in vitro*, como el de Bredbenner *et al*. ^22^, simularon defectos periimplantarios en costillas de bovino, ya que la densidad ósea y la relación entre el hueso cortical y el esponjoso son similares a los de la mandíbula humana. 

Asimismo, Dave *et al.*^23^ demostraron que las radiografías periapicales convencionales fueron significativamente mejores para detectar radiolucencias periimplantarias que las TCHC, cuando se compara la ausencia del defecto periimplantario con la presencia del defecto (0,35 mm), y que, a medida que el defecto óseo periimplantario aumenta de tamaño, no existe una diferencia significativa en la precisión del diagnóstico. Nuestro estudio coincidió con el de Dave *et al.*, los cuáles encontraron que la radiografía periapical digital es un instrumento con un alto grado de significancia en la detección de defectos óseos periimplantares, en comparación con la tomografía ^23^.

Del mismo modo, Dave *et al*. ^23^ y Sewerin *et al*. ^24^ refieren que las radiografías intraorales son las más usadas en odontología y, por ese motivo, los examinadores tienen más experiencia en su interpretación, en comparación con las TCHC. En nuestro estudio, sin embargo, todos los examinadores estaban también familiarizados con el manejo de TCHC para la planificación de implantes dentales. Asimismo, los resultados del estudio de Dave *et al*. demuestran que la radiografía tiene una sensibilidad y especificidad excelentes en la detección de defectos óseos periimplantarios, y que funciona significativamente mejor que la TCHC en ese sentido.

Finalmente, en base a lo analizado, y reconociendo que se debe profundizar en esta línea de investigación, este estudio enfatiza que la RDD se desempeñó significativamente mejor que la TCHC.

## CONCLUSIONES

La RDD se desempeñó significativamente mejor que la TCHC y los resultados no apoyan el uso rutinario de TCHC como reemplazo de la RDD para el diagnóstico de defectos óseos periimplantarios en una fase inicial. Este hallazgo se aplica a los defectos que son circunferenciales y no a los que se encuentran únicamente en las superficies vestibular o lingual/palatina.

## Fuente de financiamiento

Autofinanciado
